# The Ablation of Envelope Protein Glycosylation Enhances the Neurovirulence of ZIKV and Cell Apoptosis in Newborn Mice

**DOI:** 10.1155/2021/5317662

**Published:** 2021-07-16

**Authors:** Yanqing Guo, Linlin Bao, Yanfeng Xu, Fengdi Li, Qi Lv, Feiyue Fan, Chuan Qin

**Affiliations:** ^1^Comparative Medicine Center, Peking Union Medical College (PUMC) and, Institute of Laboratory Animal Sciences, Chinese Academy of Medical Sciences (CAMS), Beijing 100021, China; ^2^Key Laboratory of Human Disease Comparative Medicine, Ministry of Health, Beijing 100021, China; ^3^Beijing Key Laboratory for Animal Models of Emerging and Reemerging Infectious, Pan Jia Yuan Nan Li No. 5, Chao Yang District, Beijing 100021, China

## Abstract

Zika virus (ZIKV) has attracted the wide global attention due to its causal link to microcephaly. In this study, two amino acid (aa) mutation (E143K and R3394K) were identified at the fourth generation (named ZKC2P4) during the serial passage of ZIKV-Asian lineage ZKC2/2016 strain in the newborn mouse brain, while another seven aa deletions in envelope (E) protein were detected in ZKC2P6. ZKC2P6 is a novel nonglycosylated E protein Asian ZIKV we first identified and provides the first direct supporting evidence that glycosylation motif could be lost during the passage in neonatal mice. To study the impact of E protein glycosylation ablation, we compared the pathogenicity of ZKC2P6 with that of ZKC2P4. The results showed that the loss of E protein glycosylation accelerated the disease progression, as evidenced by an earlier weight loss and death, a thinner cerebral cortex, and more serious tissue lesions and inflammation/necrosis. Furthermore, ZKC2P6 exhibited a greater ability to replicate and caused severer cell apoptosis than that of ZKC2P4. Therefore, the ablation of E glycosylation generally enhances the neurovirulence of ZIKV and cell apoptosis in newborn mice.

## 1. Introduction

Zika virus (ZIKV), a member of the mosquito-borne Flaviviridae family, was originally isolated from a rhesus monkey from the Zika forest in 1947 [1]. Phylogenetic analyses have revealed two main ZIKV lineages, African and Asian, and the contemporary epidemics have been traced to the Asian lineage [2]. Currently, ZIKV has been causally linked to microcephaly during pregnancy and thus evolved into a new public health threat [3]. The genome of ZIKV is a positive-sense RNA that contains one open reading frame encoding a polyprotein, which can be cleaved into three structural proteins, including capsid (C), precursor membrane (PrM), and envelope (E), and seven nonstructural (NS) proteins [4, 5]. The E protein is involved in many crucial steps of the viral lifecycle, including the interactions with cellular surface attachment factors and receptors, the fusion of viral and cellular membranes, virus assembly, and liberation. To date, three types of mouse models, including the embryonic microcephaly model [6], the neonatal mouse model [7], and the adult mouse model lacking the IFN receptor (Ifnar^−/−^ model) [8], were used to study the virulence of ZIKV. Although these three models have some degree of immune deficiency, the developmental state of the first two models is closer to that of human microcephalic fetus. The glycan on N154 of E protein projects from the viral surface and may be directly involved in cell receptor binding [9]. Glycosylation site deficiency can be achieved in two ways: the substitution of a single key aa (an aa in the glycosylation motif NDT) or the deletion of a few aa spanning the N154 site. For example, the loss of the N154 glycosylation site via a deletion has been observed in the African ZIKV [4, 10] while the glycosylation site motif was lost because of a 1-aa substitution in dengue virus (DV) and ZIKV [4]. The loss of the N154 glycosylation site has been observed in some flaviviruses, and this phenomenon may be related to passaging in the mouse brain, but no direct evidence exists to support this hypothesis [4, 10].

In the present study, we obtained a novel nonglycosylated E protein Asian ZIKV, named ZKC2P6, via spontaneous amino acid deletion during neonatal Balb/c mice brain passage. The ablation of envelope protein glycosylation of ZKC2P6 enhanced its replication ability and induced severer histological lesions, inflammation/necrosis, and more cellular apoptosis in newborn mice. This finding also provides the first direct supporting evidence that glycosylation motif could be lost during brain passage in neonatal mice.

## 2. Materials and Methods

### 2.1. Cells and Viruses

Human neuroblastoma cell line SH-SY5Y (ATCC CRL-2266) was maintained in MEM containing 15% fetal bovine serum (FBS) (Gibco, USA) at 37°C with 5% CO_2_. The Vero African green monkey kidney epithelial cell line (ATCC CCL81) was cultured in DMEM supplemented with 10% FBS. The ZIKV strain ZKC2/2016 (KX253996) was isolated from an imported case [11]. ZKC2P4 and ZKC2P6 were derived from ZKC2/2016 after four and six passages in suckling mice, respectively. The supernatant of the solution containing homogenized mouse brains at 5 days postinfection (DPI) was harvested, titrated using a real-time reverse transcription (RT)-PCR assay, and then stored at -80°C. ZIKV strains were purified three times using a plaque assay on Vero cells as described below. Studies with ZIKV were conducted under BSL2 and animal BAL3 conditions in the Institute of Laboratory Animal Sciences (ILAS) with Institutional Biosafety Committee approval.

### 2.2. Plaque Assay

ZIKV samples were serially diluted tenfold for 6 times in DMEM, and 1 ml virus in each dilution was added to Vero cell monolayers in 6-well plates and was then incubated at 37°C with 5% CO_2_. After a 1 h incubation, the supernatants were discarded and washed with phosphate buffered saline (PBS). Then, 2 ml of DMEM containing 1% low-melting-point agarose (Sigma) was added to each well, the plates were incubated at 37°C with 5% CO_2_ for 4 days, and the monoclonal virus was selected and amplified in 12-well plates.

### 2.3. Reverse Transcription PCR (RT-PCR)

The virus RNA was extracted from preserved supernatant using an RNeasy Mini Kit (QIAGEN, Germany); then, the viral genome was amplified using the PrimeScript One-Step RT-PCR Kit (TaKaRa, China). Primers in this study were designed using the Oligo software (Supplemental Table 1). ZIKV RNA copies were determined using a specific probe (5′-FAM-CGGCATACAGCATCAGGTGCATAGGAG-MGB-3′) and primer set (ZIKA 835: TTGGTCATGATACTGCTGATTGC; ZIKA 911c: CCTTCCACAAAGTCCCTATTGC) [12]. Following the manufacturer's protocol, 25 *μ*l reactions of the QuantiTect Probe RT-PCR Kit with 5 *μ*l of RNA template were used to perform qRT-PCR assays using an ABI Step One Plus System. ZIKV RNA (535-1211), which was transcribed using the MEGAshortscript T7 Transcription Kit in vitro and the MEGAclear Transcription Clean-Up Kit (Invitrogen, USA), was used as the RNA standard to determine the number of viral copies in the samples.

### 2.4. Genetic, Phylogenetic, and Structural Analyses

ZIKV genomes were amplified via RT-PCR assays using the corresponding primers (Supplemental Table 1), assembled using the SeqMan software in the DnaStar package, and then aligned using the MEGA 5.05 software. Phylogenetic trees were generated using the maximum-likelihood method with default settings implemented by the MEGA 5.05 software. The structure of the E protein was predicted using the Swiss-Model online tool (https://swissmodel.expasy.org/), while the structure of glycan was built using ChemBioDraw Ultra. The structure around N154 in the E protein was interpreted using the PyMol software. The superposition of the predicted structure was conducted using the Chimera software.

### 2.5. Virus Replication Kinetics

The cell culture supernatants were discarded, and virus-medium (containing 1.5 × 10^7^ viral copies) and 0.5 mL of fresh medium (MEM containing 4% FBS) were added when the SH-SY5Y cells reached 90% confluence in 12-well plates. The infected cells were cultured for indicated time points (0 h, 6 h, 12 h, 24 h, 48 h, and 72 h), and the corresponding supernatants were collected. Virus loads in supernatants were determined using quantitative RT-PCR.

### 2.6. Animal Experiments

One-day-old Balb/c mice were intracranially inoculated using 25 *μ*l of ZKC2P4 or ZKC2P6 virus stocks (containing 1.5 × 10^7^ viral copies) or uninfected supernatant of the solution containing the ground brains from age-matched mice. The animals were observed and weighed at 0, 1, 3, 5, and 7 DPI. The brains and spinal cords were collected at 3 and 5 DPI for histopathological analysis and the determination of viral copies. Mouse brains were collected at 5 DPI for western blot. The protocols were approved by the Animal Use and Care Committee of the Institute of Laboratory Animal Science, Peking Union Medical College (Permit Number: ILAS-BLL17007). The animal experiments were conducted following the guidelines for animal welfare of the World Organization for Animal Health.

### 2.7. Histopathology and Immunofluorescence

Tissues for paraffin embedding were processed routinely, and the 4 *μ*m thickness sections were stained with Nissl staining. The Nissl-stained slides were viewed by light microscopy or Nano Zoomer S60. For immunofluorescence detection, the sections were incubated in 0.5% Triton X-100 in PBS for cell membrane penetration, in 0.01 M sodium citrate for antigens reparation, and blocked at RT for 1 h in 10% BSA (Sigma). The sections were then incubated in the primary antibody at 4°C overnight, washed with PBS, and incubated in the secondary antibody at RT for 1 h. After washing with PBS, the slides were finally stained with DAPI (Abcam, UK) for 5 min and then were viewed with a Leica Microsystems or Nano Zoomer S60 instrument. The antibodies used for immunofluorescence were as follows: Z6 provided by George Fu Gao (1 : 250) (Ma et al., 2016), anti-cleaved caspase-3 rabbit mAb (9664 s, 1 : 500; CST), anti-GFAP rabbit mAb (80788, 1 : 500; CST), anti-Sox2 rabbit mAb (23064, 1 : 500; CST), anti-NeuN rabbit mAb (24307, 1 : 500; CST), fluorescein-conjugated Affinipure goat anti-human IgG (H+L) (ZF0308, 1 : 500; ZSGB-BIO, China), and Alexa Fluor 594-conjugated anti-rabbit IgG (H+L) (8889 s, 1 : 1000; CST).

### 2.8. Western Blot

The western blot was performed as previously reported [13]. Briefly, the harvested brains were washed with cold PBS and fully lysed with RIPA buffer. The lysed brains were placed on the ice for 30 min and then were centrifuged for 30 min at 13,000 rpm at 4°C to remove tissue debris. The protein concentration was determined using the BCA method. Proteins were analyzed under denaturing conditions in 12% SDS-PAGE and transferred onto a nitrocellulose filter membrane. The membranes were blocked with 5% skim milk in TBST (TBS with 0.1% Tween 20) buffer for 1 h and then incubated overnight at 4°C with antibodies against PARP (9542 s, 1 : 1000; CST) and *β*-actin (4970 s, 1 : 1000; CST). After washing with TBST, the membranes were further incubated with the second antibody conjugated to HRP (7074 s, 1 : 5000; CST) for 1 h at room temperature and then were washed with TBST. The blots were exposed using ECL western blotting detection reagent and scanned using the Bio-Rad Quantity one Program.

### 2.9. Statistical Analysis

All the data were analyzed using GraphPad Prism 5.0 or SPSS 16.0 software. Survival curves were analyzed using the log-rank (Mantel-Cox) test. The weight changes were analyzed using a repeated measures ANOVA. Other results were analyzed using Student's unpaired *t*-test or a one-way ANOVA with Tukey's multiple comparison test. The data are presented as the means ± SD. The *p* < 0.05 was considered statistically significant.

## 3. Results

### 3.1. Discovery of the Envelope Protein Glycosylation Ablation Zika Virus

**Figure 1:**
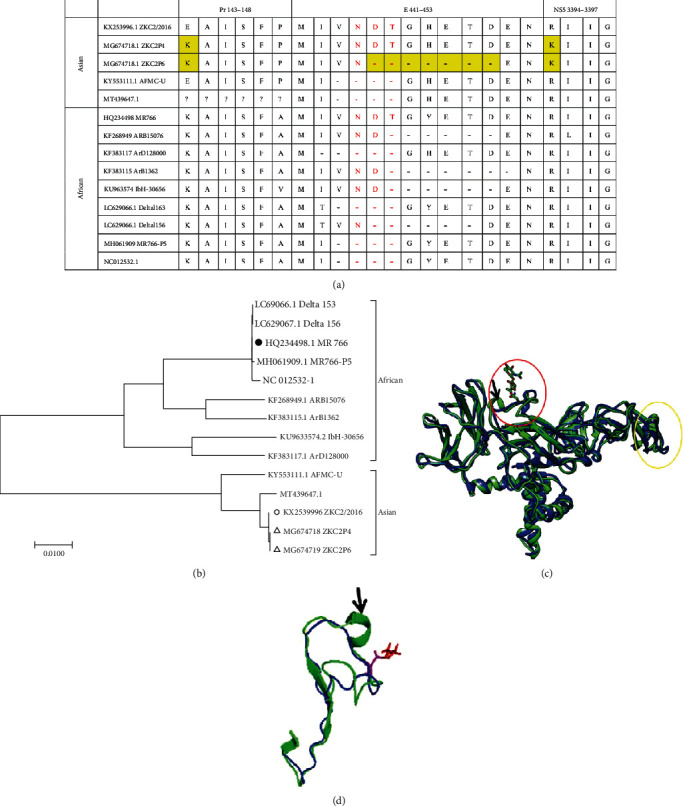
Mutation, phylogenetic, and structural analyses of the ZKC2P4 and ZKC2P6 strains. (a) The aa mutation (highlighted yellow) of the ZKC2P4 and ZKC2P6 strain are aligned to the corresponding aa of other ZIKV strains. “-” indicates an aa deficiency. Red indicates the N154 glycosylation site. (b) Phylogenetic tree constructed based on the nucleic acids of the complete open reading frame by the maximum-likelihood algorithm in the MEGA v5.05 software. “●” indicates the African lineage reference strain; “○” indicates the Asian lineage reference strain; “△” indicates the strains used in this study. (c) Superposition of the artist-rendered models of E proteins in the ZKC2P4 (green) and ZKC2P6 (blue). Red circle indicates the loop region surrounding the glycosylation site, and yellow circle indicates the glycan loop. (d) Superposition of the loop region surrounding the glycosylation site (140 to 177 in ZKC2P4 and 140 to 170 in ZKC2P6) of the E protein. The stick structure represents the glycan of the ZKC2P4 strain, and the alpha-helix is indicated by the black arrow.

Similar to other flaviviruses, ZIKV can be passaged in the brain of newborn mice. In this study, we conducted the serial passage of ZKC2/2016, which was isolated from an imported case in China [11], in one-day-old neonatal mice, and then experimentally isolated a ZIKV strain with substantial neurovirulence. E143K and R3394K were found by sequencing and alignment in the fourth generation, and seven aa (from 445 to 451) were further deleted in the fifth to the eighth generations (Figure 1(a)). The fourth and sixth generations were purified three times using the plaque assay method and were named strains ZKC2P4 (MG674718) and ZKC2P6 (MG674719), respectively. As of May 2021, 66 other strains with a loss of N-linked glycosylation from aa deletion have been identified in 945 complete ZIKV sequences in GenBank. In our study, 12 representative strains were shown (Figure 1(a)) and phylogenetic analysis revealed that the ZKC2P6 strain is a novel nonglycosylated E protein strain occurring via aa deletion in the Asian lineage (Figure 1(b)). Additionally, ZKC2P6 provides the first direct supporting evidence that glycosylation motif could be lost during the passage in neonatal mice, which is consistent with the clinical findings of another two similar but not identical amino acid deletions [14, 15].

### 3.2. Structure Prediction of the E Protein

The deletion of a few aa spanning the N154 site of the ZKC2P6 strain may impact the E protein structure. To further determine its effect, the structure of the E protein of ZIKV (PDB codes: 5IZ7.2.H and 5IZ7.2.1) was used as a template for the E proteins of ZKC2P4 and ZKC2P6, respectively. The superposition of the ZKC2P4 and ZKC2P6 E proteins and the region spanning the N154 site (red in Figure 1(a)) showed that the loss of N-linked glycosylation in the E protein of the ZKC2P6 strain sharply impacts the local alpha-helix (Figures 1(c) and 1(d)). The E protein is responsible for virus entry by interacting with cell surface receptors and represents an important determinant of viral pathogenesis [16]. Therefore, the change in the ZKC2P6 E protein structure may impact viral pathogenesis.

### 3.3. Envelope Protein Glycosylation Ablation Enhances the Neurovirulence of ZIKV in Neonatal Mice

**Figure 2:**
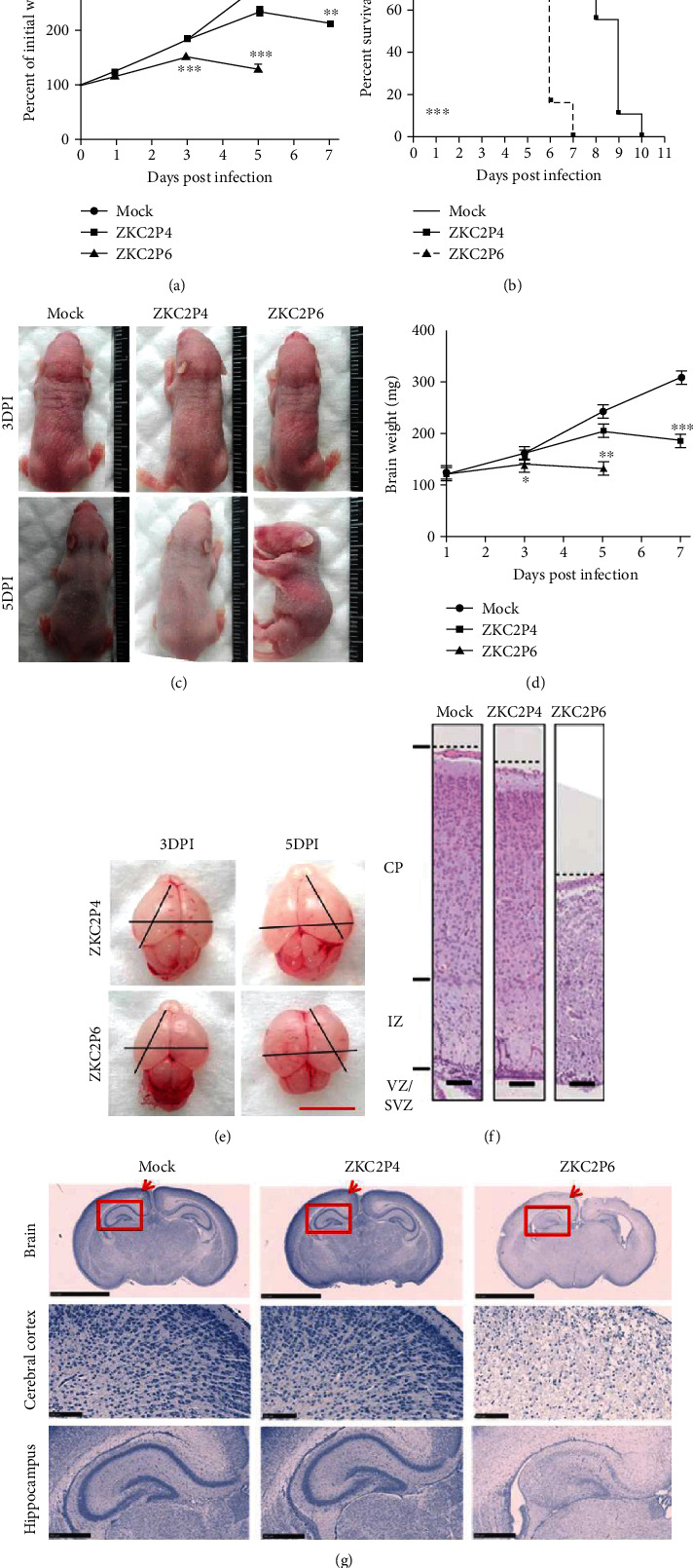
Envelope protein glycosylation ablation enhances the neurovirulence of ZIKV in neonatal mice. (a) The changes of body weight. *N* = 18, ^∗∗∗^*p* < 0.001. (b) Survival rate in neonatal mice after 1.5 × 10^7^ viral copies of ZKC2P4/ZKC2P6 infection or the uninfected supernatant injection. *N* = 18, ^∗∗∗^*p* < 0.001. (c) Body length and posture of neonatal mice at 3 and 5 DPI. *N* = 6. (d) Brain weight of neonatal mice. *N* = 6, ^∗^*p* < 0.05, ^∗∗^*p* < 0.01, and ^∗∗∗^*p* < 0.001. (e) Microcephalic brains from mice in the ZKC2P4 and ZKC2P6 groups. Black bars represent the brain width or cerebral cortex length, scale bar: 0.5 cm. (f) Brain cortices and (g) histopathological analysis of the cerebral cortex, hippocampus, and cervical spinal cord in each group at 5 DPI. Nissl staining, scale bar: 100 *μ*m.

To determine the effect of E protein glycosylation ablation for the neurovirulence of ZIKV in neonatal mice, we compared the in vivo data of ZKC2P6 with those of ZKC2P4 in one-day-old Balb/c mice that underwent intracerebral injection inoculation. Notably, ZKC2P6-infected mice presented an earlier weight loss (Figure 2(a)), emergence of symptoms, including stomach emptying, motor weakness, and hind limb paralysis (data not shown), and death (Figure 2(b)) than did ZKC2P4. There was no obvious difference in body length and posture at 3 DPI, while significant differences were found at 5 DPI (Figure 2(c)). An inspection of the Balb/c mice at 3 and 5 DPI showed that the ZKC2P6 infection led to obviously microcephalic brains (Figures 2(d) and 2(e)). In addition, we noticed a thinner cortex in the ZKC2P6-infected brains (Figure 2(f)). The microcephaly phenotype caused by ZKC2P6 is similar to that observed in the study by Li et al. [6]. Histological lesions and inflammation/necrosis in the cerebral cortex, hippocampus, cervical spinal cord, cerebellum, and brain stem were observed at earlier time points and more severe after ZKC2P6 infection (Figure 2(g) and Table 1). More death of neurons and glial cells was also observed in the cerebral cortex, hippocampus, and cervical spinal cord in the ZKC2P6 group (Figure 2(g)). Meanwhile, edemas were found in the brains and cervical spinal cords of the ZKC2P6-infected group, while no lesions in the kidney were found in any of the three groups (Table 1). Therefore, envelope protein glycosylation ablation in ZKC2P6 is more virulent to newborn mice than ZKC2P4.

### 3.4. Envelope Protein Glycosylation Ablation Significantly Enhances ZIKV Replication

**Figure 3:**
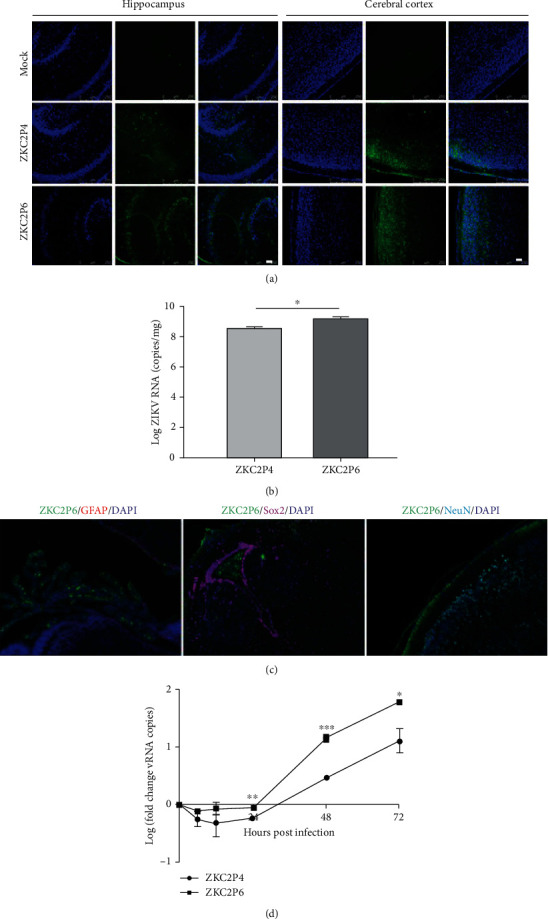
Envelope protein glycosylation ablation of ZIKV enhances viral replication in the brains of neonatal mice. (a) ZIKV viral particles (green) in the cerebral cortex and hippocampus at 5 DPI. Scale bar: 50 *μ*m. (b) Qualification of ZIKV in the brains of one-day-old Balb/C mice at 5 DPI after 1.5 × 10^7^ viral copies of ZKC2P4/ZKC2P6 infection. *N* = 6, ^∗^*p* < 0.05. (c) Colocalization of ZKC2P6 with NeuN+ neurons, GFAP+ glial cells, and Sox2+ neural stem cells using immunofluorescence. (d) Growth curves of ZKC2P4 and ZKC2P6 in SH-SY5Y cells after 1.5 × 10^7^ viral copy infection. *N* = 3, ^∗∗^*p* < 0.01.

To study the effect of E glycosylation ablation for ZIKV replication, the ZIKV particles in the cerebral cortex and hippocampus of neonatal mice at 5 DPI were determined and the result showed that the virus loads were significantly higher in the ZKC2P6 group than that of the ZKC2P4 (Figure 3(a)). The quantification of ZIKV loads in the brain further verified the enhanced replication capability of ZKC2P6 (Figure 3(b)). Similar results were also observed in the cerebral cortex of neonatal mice at 3 DPI and hippocampus of neonatal mice (data not shown). Meanwhile, detailed location analysis in normal neural cells of neonatal mice suggested that ZKC2P6 did not infect the NeuN+ neurons, GFAP+ glial cells, and Sox2+ neural stem cells, as no colocalization of the virus was detected in the corresponding cells (Figure 3(c)). Furthermore, the enhanced ZIKV replication after E glycosylation ablation was further verified in human neuroblastoma cell line SH-SY5Y at 24, 48, and 72 h postinfection (Figure 3(d)). Therefore, E glycosylation ablation significantly enhances ZIKV replication in our study.

### 3.5. Envelope Protein Glycosylation Ablation Significantly Enhances the Apoptosis of Nerve Cells in the Brain

**Figure 4:**
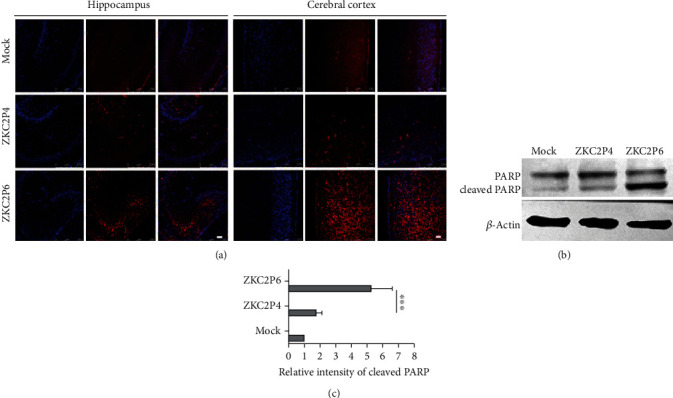
Envelope protein glycosylation ablation of ZIKV enhances the apoptosis of nerve cells. (a) Apoptosis of nerve cells in the cerebral cortex and hippocampus at 5 DPI. Red indicates the cleaved caspase-3 after immunofluorescence assay. Scale bar: 50 *μ*m. (b, c) The expression of cleaved PARP in the brains at 5 DPI. *N* = 6, ^∗∗∗^*p* < 0.001.

ZIKV infection can lead to caspase-3 (Cas3) activation in both neural precursor cells and embryonic mouse brain [17]. To determine whether ZKC2P6 accelerates the apoptosis of nerve cells in the brain, immunofluorescence for cleaved caspase-3 and western blotting for the cleaved PARP, one of the main cleavage targets of Cas3, were detected. We found that ZKC2P6 induced more apoptosis (cells positive for the cleaved Cas3) in the cerebral cortex and hippocampus of neonatal mice at 5 days postinfection of ZKC2P6 compared with the ZKC2P4 and mock groups (Figure 4(a)). Furthermore, the protein level of cleaved PARP (Figures 4(b) and 4(c)) in the ZKC2P6 group was higher than that of ZKC2P4 and mock groups. These results hinted that E glycosylation ablation significantly enhances the apoptosis of nerve cells in the brain.

## 4. Discussion

Over the past decades, the loss of the glycosylation site has been observed in many flaviviruses, such as WNV [18], DV [19], KJV [20], and ZIKV [4]. Here, we purified two ZIKV strains (ZKC2P4 and ZKC2P6) with spontaneous aa substitution in PrM and NS protein region during the serial passage of ZKC2/2016 in the mouse brain and further observed 7 aa deletions spanning the glycosylation motif in ZKC2P6 stain. To our knowledge, the ZKC2P6 strain is the first Asian strain with a glycosylation motif deficiency spontaneously produced via aa deletion during brain passage in newborn mice (Figure 1(b)). Envelope protein glycosylation mediated Zika virus pathogenesis [21]. In the present study, the ablation of E glycosylation in ZKC2P6 enhanced its capability of viral replication and neurovirulence, leading to histological lesions, inflammation/necrosis, cellular apoptosis, and death in the mouse brain.

Except for ~10 amino acids (aa) surrounding the N-linked glycosylation site (N154), the ZIKV structure is similar to other flavivirus structures [9, 22]. In this study, 7 aa spanning the N154 site are deleted in ZKC2P6, while similar but not the same strains with aa deletion spanning the N154 site (MT439647, KY553111, KF268949, KF383117, KF383115, KU963574, LC629066, LC629067, MH061909, and NC012532) were previously recorded in the complete ZIKV sequences from GenBank (Figure 1(a)). Our results during newborn mouse brain passage further provided the direct evidence to support this hypothesis that glycosylation motif may be lost during ZIKV passage histories [4]. Six to nine aa near the glycosylation site in E protein of ZIKV, which is longer than that in several (but not all) other flaviviruses, leads to the projection of the glycan on N154 from the viral surface [9]. The deletion of this seven aa in our study was predicted to influence the structure of the glycan loop (Figures 1(c) and 1(d)) and may influence the spatial structure of the E protein dimer for the existence of the glycan loop [9].

The E protein is responsible for virus entry and impacts viral fitness, infectivity, replication, and virulence in many flaviviruses [23]. In our study, envelope protein glycosylation ablation enhanced the ZIKV replication and neurovirulence in neonatal mice. Mossenta et al. proved that N-linked glycosylation at Asn-154 in ZIKV influences viral assembly and infectivity in vitro [24], while Fontes-Garfias and his colleagues demonstrated that E glycosylation was necessary for the ZIKV infection in Ifnar1-/- mice and mosquito hosts but did not significantly affect neurovirulence in newborn mice [25]. However, we observed that E protein glycosylation ablation enhanced the neurovirulence of ZIKV in newborn mice, which represents a discrepancy with the results by Fontes-Garfias et al. We speculated three possible reasons may explain this discrepancy. First, the CD-1 and Balb/C mice may have different sensitivities to ZIKV; second, the neurovirulence of ZKC2P6 in newborn mice may be related to aa near the N154 site besides glycan on N154, while the FSS13025 strain lost the glycosylation motif through a single aa substitution. Third, other 19 aa of FSS13025, including an important pathogenic site at position 139 [16], were different from the corresponding aa of ZKC2P6. Moreover, the hepacivirus genera HCV also belong to the family of the Flaviviridae, though not the flaviviruses [26]. Our results about the enhanced ZIKV replication, neurovirulence, tissue lesions, inflammation/necrosis, and apoptosis after E protein glycosylation ablation are in line with the fact that the deletion of N-glycosylation sites of HCV E1 envelope protein enhances specific cellular and humoral immune responses [27]. In the present study, we also verified that ZKC2P6 did not infect the GFAP+ glial cells, NeuN+ neurons, and Sox2+ neural stem cells (Figure 3(c)), which is consistent with the conclusion that ZIKV did not infect NeuN+ neurons and GFAP+ glial cells in normal adult human brain tissues [28]. However, ZIKV in Sox2+ and NeuN+ cells in the subventricular zone and dentate gyrus of the adult mouse brain were quantified in another study [29]. Meanwhile, consistent with the enhanced infectivity, envelope protein glycosylation ablation of ZKC2P6 predictively induced apoptosis of nerve cells in the brain in our study (Figure 4).

In conclusion, we identified a novel ZIKV strain, named ZKC2P6, that is the first Asian strain with a glycosylation motif deficiency caused by aa deletion during newborn mouse brain passage. Furthermore, we revealed that ZKC2P6 exhibited a greater ability to replicate and caused severer neurovirulence and cell apoptosis in newborn mice.

## Figures and Tables

**Table 1:** Lesions of cervical spinal cord, kidney, and parts of brain on 3 and 5 DPI.

DPI	Group	Cerebral cortex	Hippocampus	Cerebellum	Brain stem	Cervical spinal cord	Kidney
3	Mock	-	-	-	-	-	-
	ZKC2P4	+	+	+	-	±	-
	ZKC2P6	++	++	+	+	±	-
5	Mock	-	-	-	-	-	-
	ZKC2P4	++	+	+	±	±	-
	ZKC2P6	+++	+++	++	+	+	-

Note: -, no lesions and inflammation/necrosis; ±, minor lesions, occasional neurons/glial cell inflammation and necrosis; +, mild lesions, a few neurons/glial inflammation and necrosis; ++, moderate lesions, more neurons/glial cell inflammation and necrosis; +++, severe lesions, large number of neurons/glial inflammation and necrosis.

## Data Availability

The data supporting the conclusions of this article are included in the article.

## Supplementary Materials

Supplementary Materials Supplemental information for this article includes one table.
