# Supplementary material for: The Ablation of Envelope Protein Glycosylation Enhances the Neurovirulence of ZIKV and Cell Apoptosis in Newborn Mice
Source: J Immunol Res. 2021 Jul 16;2021:5317662. doi: 10.1155/2021/5317662 (PMC8302398; doi:10.1155/2021/5317662)
Supplement: Supplementary Materials — Supplemental information for this article includes one table. [file 5317662.f1.docx]

# The Ablation of Envelope Protein Glycosylation Enhances the Neurovirulence of ZIKV and Cell Apoptosis in Newborn Mice

Yanqing Guo^1,2,3^, Linlin Bao^1,2,3^, Yanfeng Xu^1,2,3^, Fengdi Li^1,2,3^, Qi Lv^1,2,3^, Feiyue Fan^1,2,3^ , Chuan Qin^1,2,3*^

1 Comparative Medicine Center, Peking Union Medical College (PUMC) &，Institute of Laboratory Animal Sciences, Chinese Academy of Medical Sciences (CAMS) Beijing 100021, China.

2 Key Laboratory of Human Disease Comparative Medicine, Ministry of Health, Beijing 100021, China.

3 Beijing Key Laboratory for Animal Models of Emerging and Reemerging Infectious, Pan Jia Yuan Nan Li No.5, Chao Yang District, Beijing 100021, China.

*Correspondence should be addressed to Chuan Qin; qinchuan@pumc.edu.cn

Supplement Table 1: Primers for Zika virus genome sequence used in this study

| Primers | Position | Sequence |
| --- | --- | --- |
| ZIKV-F0 | 1-23 | AGTTGTTGATCTGTGTGAATCAG |
| ZIKV-R0 | 456-434 | GCAGGAGGCCAACAATTCCGACA |
| ZIKV-F1 | 134-158 | AGGATTCCGGATTGTCAATATGCTA |
| ZIKV-R1 | 1494-1478 | GTGAATTGGGCGTTATCTCA |
| ZIKV-F2 | 1315-1334 | TGGTGACATGCGCTAAGTTT |
| ZIKV-R2 | 2709-2685 | CCCCTTCTACTGATCTCCACATGAT |
| ZIKV-F3 | 2522-2542 | GGAGACGAGATGCGGTACAGG |
| ZIKV-R3 | 3874-3898 | ATCGCAGTTTGCAAAAGACACGAGG |
| ZIKV-F4 | 3744-3769 | AAATGAACACTGGAGGAGATGTAGC |
| ZIKV-R4 | 5116-5094 | CCTTGGGTGATGGCACTAACATA |
| ZIKV-F5 | 4948-4969 | TGCCCGGAATATTTAAGACAAA |
| ZIKV-R5 | 6344-6320 | CGGCACACTGTCTTCCATTATGGTG |
| ZIKV-F6 | 6199-6223 | GGAAGACCTTTGTGGAACTCATGAA |
| ZIKV-R6 | 7590-7566 | TGTTACACAGTGAAGTGGCTGTAGA |
| ZIKV-F7 | 7418-7442 | AAAGATGGGACAGGTGCTACTCATA |
| ZIKV-R7 | 8812-8789 | AACCAGGAAGAGACCATGCTCATA |
| ZIKV-F8 | 8682-8704 | GTCACAGGAATAGCCATGACCGA |
| ZIKV-R8 | 10075-10051 | CCCTTTCCATGGATTGACCAGGTAG |
| ZIKV-F9 | 9858-9878 | CGCCACCAAGATGAACTGATT |
| ZIKV-R9 | 10807-10787 | AAGACCCATGGATTTCCCCAC |
